# Case report: Pacemaker lost capture after acute myocardial infarction in a patient with left circumflex coronary artery occlusion

**DOI:** 10.3389/fcvm.2022.993903

**Published:** 2022-12-06

**Authors:** Zhihong Wu, Jianjun Tang, Qingyi Zhu, Lin Hu, Zhenjiang Liu, Xuping Li, Qiming Liu, Shenghua Zhou, Mingxian Chen

**Affiliations:** Department of Cardiology, The Second Xiangya Hospital of Central South University, Changsha, China

**Keywords:** acute myocardial infarction, pacemaker, failure capture, sudden cardiac death, arrest

## Abstract

A 71-year-old female with a dual-chamber pacemaker presented to our hospital complaining of repeated chest pain. She was diagnosed with unstable angina. On day 7, the patient suddenly suffered cardiopulmonary arrest due to an inferior ST segment elevation myocardial infarction (STEMI). Pacemaker lost capture was suspected and was later confirmed by a pacemaker check with a high pacing threshold and a low sensing parameter. Emergency coronary angiography revealed that a large filling defect remained due to an extensive thrombus in the proximal left circumflex (LCX) with thrombolysis in myocardial infarction (TIMI) grade 2 flow, and then a repeat thrombus aspiration was performed. After reperfusion, the parameters of the right ventricular lead were gradually returned. We conclude that the loss of the right ventricular lead pacing occurred in this case of acute coronary syndrome (ACS) induced by an LCX thrombus due to an LCX supplying the right ventricular septal.

## Introduction

There are several causes that contribute to the loss of a permanent pacemaker capture, such as battery depletion, lead dislodgement, and circuit problems. However, malfunction of a ventricular lead of a dual-chamber pacemaker following an acute myocardial infarction (AMI) is rare (1). There are few reports describing the pacemaker lost atrial capture as a consequence of an AMI (2–4). Herein, we report the rare case of failure pacing following an acute inferior wall myocardial infarction in a patient with an occlusion of the left circumflex (LCX) that supplied the right ventricular septal.

## Case report

A 71-year-old female presented to our hospital complaining of repeated chest pain. Her past history included coronary heart disease and sick sinus syndrome (SSS). Six years ago, she was first admitted to our hospital and diagnosed with unstable angina. Then, coronary angiography revealed a severe stenosis of the left anterior descending (LAD), and a stent was implanted into the LAD. One year ago, the patient was admitted to our hospital because of syncope. She was diagnosed as having SSS with 7.97 seconds of asystole and then received a dual-chamber pacemaker (Medtronic Inc., Minneapolis, MN, USA) implantation.

On admission, her heart rate was 76 bpm (beats per minute) and her blood pressure was 131/67 mmHg. Transthoracic echocardiography (TTE) showed a normal left ventricular size and function without regional wall motion abnormalities. Electrocardiogram (ECG) on admission shows a sinus rhythm at 76 bpm, normal conduction, and low amplitude of T wave in multiple leads. According to the ischemic symptom and previous history, the patient was diagnosed as having unstable angina. The concentration of hypersensitive cardiac troponin T (TnTsh, upper limit of normal = 14 pg/ml) was at a normal level (9.64 pg/ml). She received a secondary prevention of coronary heart disease. After admission, we confirmed that her pacemaker was functioning normally (a pacing threshold of 0.6 V @0.40 ms, a sensing threshold of 11.3–13.1 mV, and a lead resistance of 820 Ω). In addition, creatine kinase (CK) and TnTsh were normal.

**Figure 1:**
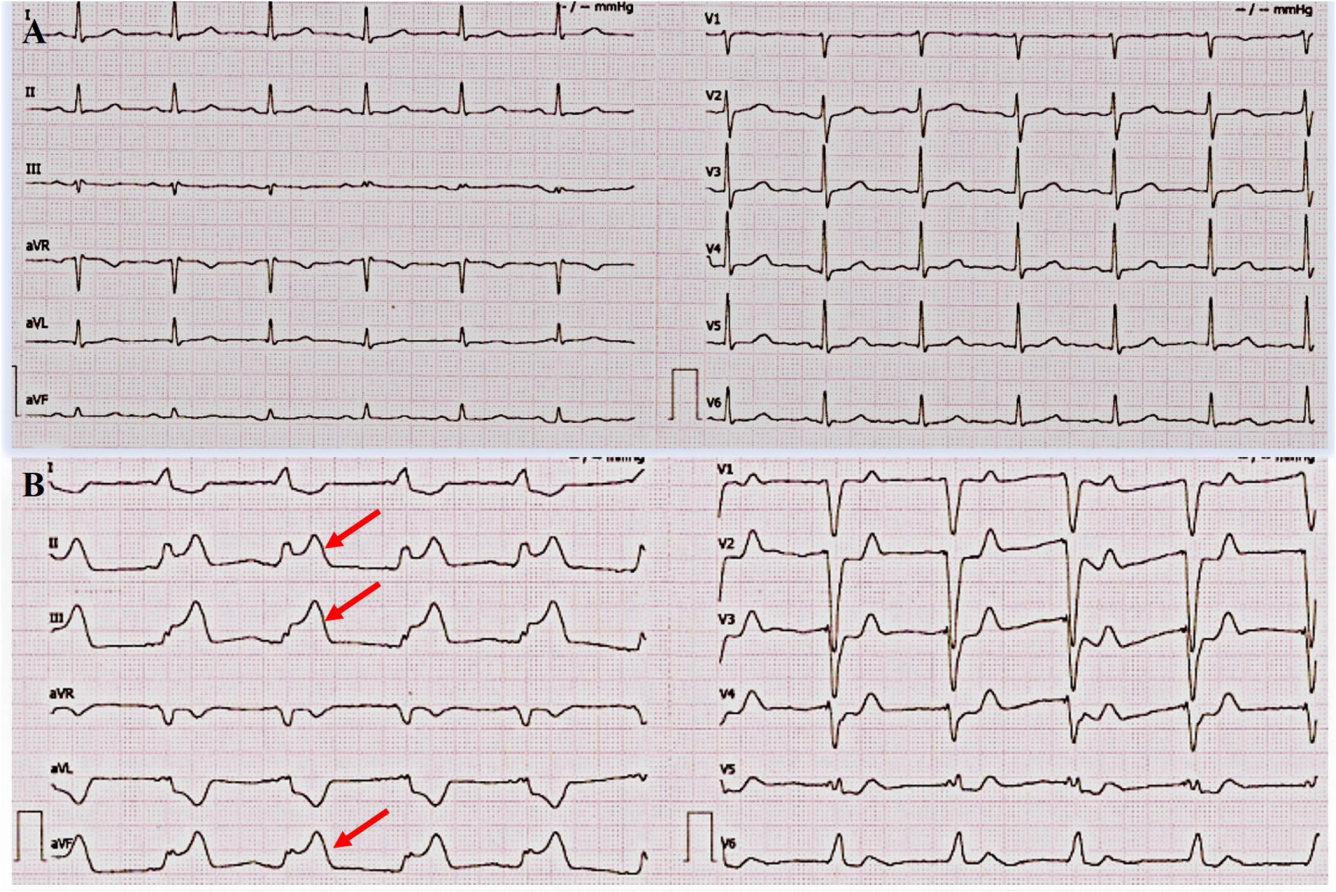
Admission electrocardiogram (ECG) and acute myocardial infarction (AMI) ECG. **(A)** ECG on admission. It showed a sinus rhythm at 76 bpm (beats per minute), normal conduction, and low amplitude of T wave in multiple leads. **(B)** ECG during AMI. It showed ST segment elevation in leads II, III, and aVF and ST segment depression in leads I, aVL, and v4-6.

**Figure 2:**
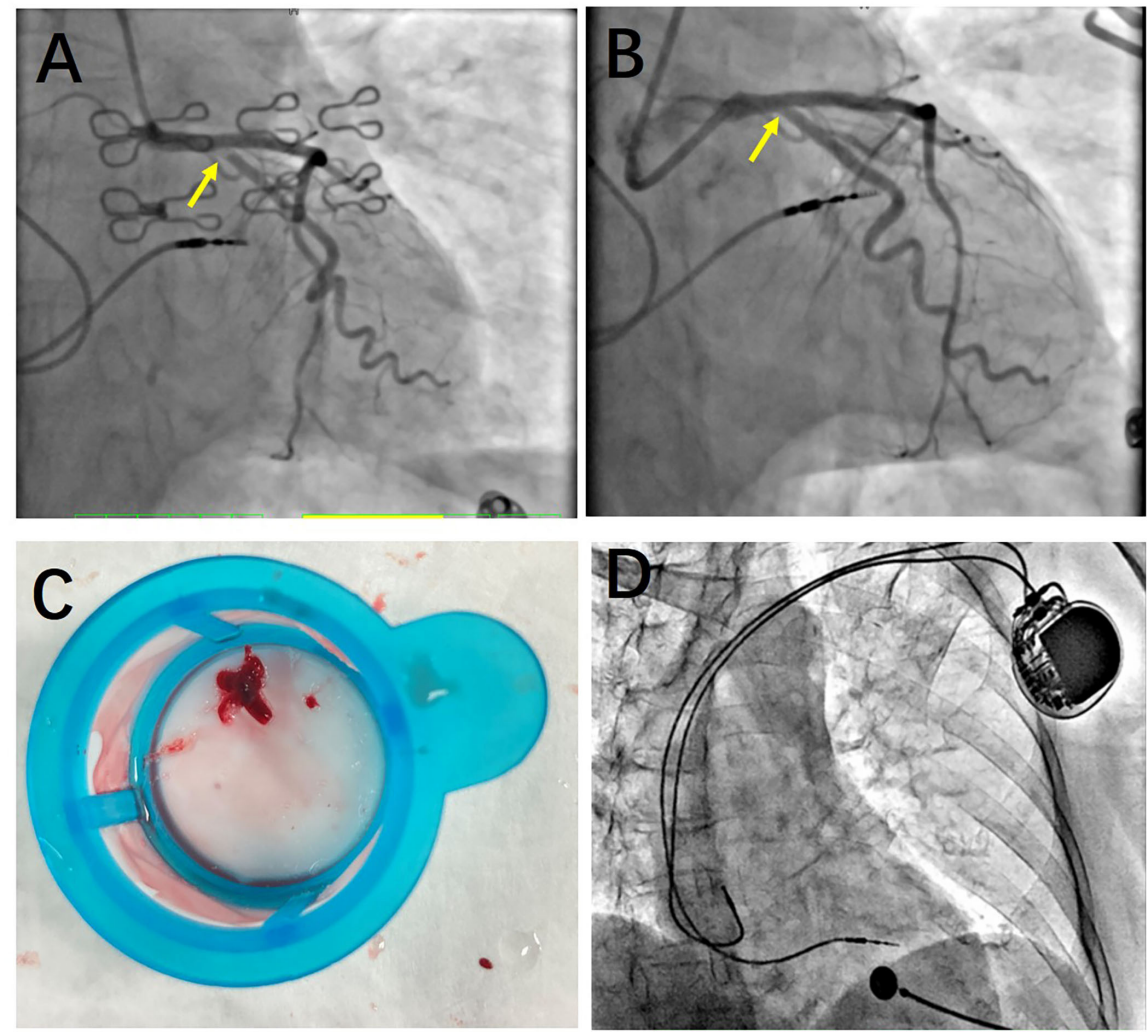
Coronary angiography. **(A)** Before thrombus aspiration, a large filling defect remained due to an extensive thrombus in the proximal left circumflex (LCX) with The Thrombolysis In Myocardial Infarction (TIMI) grade 2 flow. **(B)** After thrombus aspiration, the large filling defect appeared. And the TIMI grade of LCX improved. **(C)** A large thrombus was presented after thrombus aspiration. **(D)** Atrial and ventricular leads of dual-chamber pacemaker were normal. No leads' dislodgement or fracture was found.

**Figure 3:**
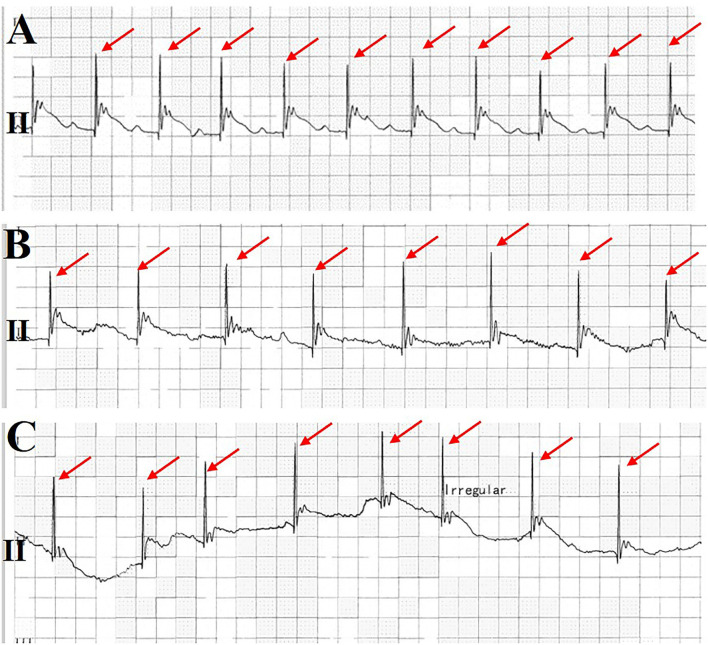
Monitoring electrocardiogram (ECG) at lead II. It was found that the pacemaker gradually lost capture before the patient lost consciousness **(A–C)**. Ventricular pacing spikes are shown in a red arrow.

On day 7, she suddenly presented with chest pain, then lost consciousness, and no carotid pulse was found. Subsequently, cardiopulmonary arrest ensued. Cardiopulmonary resuscitation (CPR) was performed immediately. ECG showed an ST segment elevation in leads II, III, and aVF and ST segment depression in leads I, aVL, and V4-6 (Figure 1). She was diagnosed as having AMI. A pacemaker malfunction was suspected and confirmed by a pacemaker check showing an increase in the pacing threshold to >10.0 V and a decrease in the sensing threshold to < 0.5 mV, despite a normal lead resistance (840 Ω). After CPR, her heart rate was 113 bpm (sinus rhythm) and her blood pressure was 128/68 mmHg (maintenance dose of norepinephrine: 1.2 mg/h). Pulse oximetry was 93% supported with mechanical ventilation. The results of arterial blood gases and electrolytes showed a pH = 7.49, a potassium concentration (3.7 mmol/L, upper limit of normal = 5.3 mmol/L), and a magnesium concentration (0.8 mmol/L, lower limit of normal = 0.78 mmol/L). The concentration of TnTsh was elevated (437 pg/ml). The results of a routine blood panel showed a normal level of hemoglobin (131 g/L). Emergency coronary angiography showed that a large filling defect remained due to extensive thrombus in the proximal left circumflex (LCX) with TIMI grade 2 flow (Figure 2). The patient underwent repeated thrombus aspirations. The TIMI grade 2 flow had improved. The pacemaker reprogramming, it was found that an increase in the pacing threshold to >5.0 V and a decrease in the sensing threshold to 2.2 mV were noticed, despite a normal lead resistance (760 Ω) (Table 1). Looking back at the ECG monitor, ventricular pacing spikes were visible and failed to capture the myocardium (Figure 3). The atrial lead functioned normally. During the follow-up, from day 1 to day 3, the parameters of ventricular lead were gradually returned. In addition, CK showed the highest value [2,467 IU/L (international units/liter)] on day 1.

**Table 1:** Summary of right ventricular lead testing before and after the acute myocardial infarction.

**Date**	**Right atrium**			**Right ventricle**		
	**P-wave (mv)**	**Threshold (V/ms)**	**Impedance (Ω)**	**R-wave (mv)**	**Threshold (V/ms)**	**Impedance**
Before AMI	>3.7	0.8@0.40	620	11.3–13.1	0.6@0.40	820
AMI	>2.8	1.0@0.40	660	< 0.5	>10@1.5	840
After thrombus aspiration	>3.1	1.2@0.40	600	2.2	>5.0@1.5	760
Day 1 after AMI	>3.5	1.0@0.40	610	3.3	2.6@0.40	780
Day 2 after AMI	>3.5	1.0@0.40	580	5.7–5.8	2.2@0.40	800
Day 3 after AMI	>3.5	0.8@0.40	590	8.1–9.2	1.2@0.40	830

The infarction was associated with a complete right ventricular function. After an acute myocardial infarction (AMI), the ventricular lead sensing and pacing parameters were found to have gradually returned.

## Discussion

There are several causes that result in the failure capture of a pacemaker, including battery depletion, lead dislodgement, circuit problems, acidosis, and hyperkalemia (5). Although pacemaker malfunction induced by ischemia is rare, there are still some previous case reports that demonstrated an atrial lead malfunction after a myocardial infarction. In these cases, the pacing threshold was obviously increased due to myocardial necrosis. However, malfunction of a ventricular lead of a dual-chamber pacemaker following an acute myocardial infarction (AMI) is rarely reported (6). This case has illustrated the fact that loss capture (GA1) of a pacemaker took place after inferior STEMI in a patient. The anterior 2/3 of the interventricular septum is supplied by the left anterior descending or LAD, while the posterior 1/3 of the interventricular septum is supplied by the right coronary artery or LCX. The ventricular lead was implanted into the posterior 1/3 of the interventricular septum. Therefore, thrombotic LCX induced ventricular lead failure capture. A recent article reported by Saadatagah et al. (7) has also presented a similar scenario on the implantable cardioverter-defibrillator (ICD) malfunction during an AMI. It showed the case of a young woman with refractory vasospastic angina who happened to display a repeated dysfunction of her implantable cardioverter-defibrillator (ICD). Coronary angiography showed severe stenosis of three vessels (7).

A few hours after the AMI, an intercellular edema occurred. It is characterized by an obvious inflammatory response with neutrophil infiltration and progressive coagulative necrosis (8). It led to an increase in the pacing threshold in this patient, and it finally, resulted in a failure capture of the right ventricular lead. After thrombus aspiration and TIMI, blood flow recovered, and the parameters of the right ventricular lead were gradually returned. We concluded that the transient ischemia of the interventricular septum at the pacer–lead interface caused a transient loss of sensing and pacing functions (9).

## Conclusion

Failure of ventricular capture of the pacemaker in an acute myocardial infarction is transient. Failure capture will reverse to normal after the timely intervention of reperfusion therapy.

## Data availability statement

The original contributions presented in the study are included in the article/supplementary material, further inquiries can be directed to the corresponding author/s.

## Ethics statement

The studies involving human participants were reviewed and approved by the Second Xiangya Hospital of Central South University. The patients/participants provided their written informed consent to participate in this study. Written informed consent was obtained from the individual(s) for the publication of this case report and of any potentially identifiable images or data included in this article.

## Author contributions

MC, ZW, and JT participated in the study design and drafted the manuscript. QZ, LH, XL, and ZL contributed to data collection. MC and QL were responsible for writing the manuscript. SZ and MC contributed to the manuscript revision. All authors contributed to the article and approved the submitted version.

## Funding

Financial support was obtained from the National Natural Science Foundation of China Grant No. 81800302 and the Provincial Natural Science Foundation of Hunan Grant Nos. 2019JJ50871 and 2022JJ30839.

## Conflict of interest

The authors declare that the research was conducted in the absence of any commercial or financial relationships that could be construed as a potential conflict of interest.

## Publisher's note

All claims expressed in this article are solely those of the authors and do not necessarily represent those of their affiliated organizations, or those of the publisher, the editors and the reviewers. Any product that may be evaluated in this article, or claim that may be made by its manufacturer, is not guaranteed or endorsed by the publisher.
